# The Role of Myeloid Cells in Hepatotoxicity Related to Cancer Immunotherapy

**DOI:** 10.3390/cancers14081913

**Published:** 2022-04-10

**Authors:** Cathrin L. C. Gudd, Lucia A. Possamai

**Affiliations:** 1 Nuffield Department of Clinical Neurosciences, University of Oxford, Oxford OX3 9DU, UK; cathrin.gudd@ndcn.ox.ac.uk; 2 Department of Metabolism, Digestion & Reproduction, Imperial College London, London SW7 2AZ, UK

**Keywords:** hepatotoxicity, myeloid cells, immunotherapy, cancer, immune-related adverse event

## Abstract

**Simple Summary:**

Immune-modulating cancer treatments have proved to be highly effective in a wide range of tumour types. They interrupt the usual communication between cells in the immune system, encouraging them to become more active in identifying and destroying cancer cells. Although these therapies are very successful in treating cancer, patients frequently experience liver injury as a side effect related to over activation of the immune system. If cancer patients develop this side effect, they need to stop their cancer therapy and be given strong immunosuppressants. Researchers are now working on understanding the mechanisms involved in the development of liver inflammation. In this review we will summarise findings identifying classes of immune cells that are of particular importance in this context and highlight ways in which we can use this knowledge to improve the safety of these new cancer drugs.

**Abstract:**

Drug-related hepatotoxicity is an emerging clinical challenge with the widening use of immunotherapeutic agents in the field of oncology. This is an important complication to consider as more immune oncological targets are being identified to show promising results in clinical trials. The application of these therapeutics may be complicated by the development of immune-related adverse events (irAEs), a serious limitation often requiring high-dose immunosuppression and discontinuation of cancer therapy. Hepatoxicity presents one of the most frequently encountered irAEs and a better understanding of the underlying mechanism is crucial for the development of alternative therapeutic interventions. As a novel drug side effect, the immunopathogenesis of the condition is not completely understood. In the liver, myeloid cells play a central role in the maintenance of homeostasis and promotion of inflammation. Recent research has identified myeloid cells to be associated with hepatic adverse events of various immune modulatory monoclonal antibodies. In this review article, we provide an overview of the role of myeloid cells in the immune pathogenesis during hepatoxicity related to cancer immunotherapies and highlight potential treatment options.

## 1. Introduction

Drug-related hepatotoxicity in the context of cancer therapy is a frequently encountered adverse event. Immunotherapy is a class of novel cancer treatment utilising the host’s immune system with the aim of re-programming effector cells to enhance their anti-tumour immune responses [1,2,3]. These include, for example: blocking immune cell checkpoints such as CTLA-4, PD-1 and its ligand PD-L1; the activation of co-stimulatory pathways (e.g., CD40, ICOS, OX40 and 4-1BB agonist); and manipulation of immunometabolism (IDO1 inhibitors). This therapeutic strategy has proven efficacy in a number of solid organ and haematological malignancies [1,4,5].

**Figure 1:**
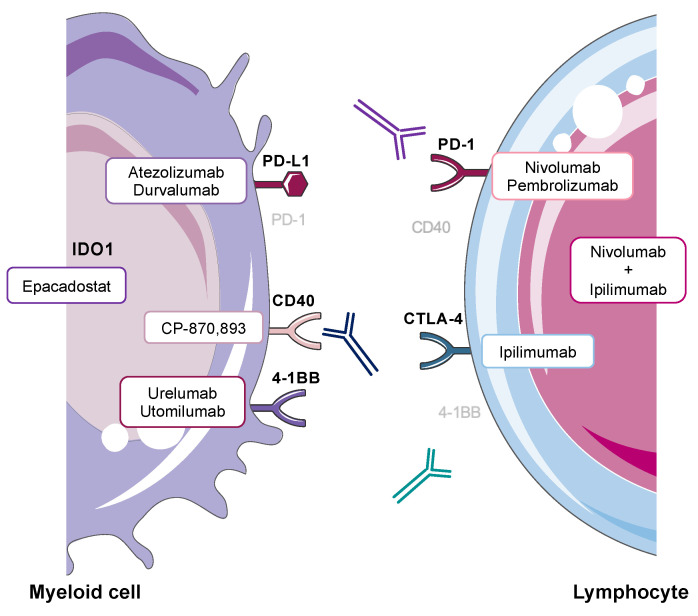
Myeloid and lymphoid cell expression of key targets of cancer immunotherapy associated with hepatotoxicity [5,18,19,20,21,22,23]. Antagonist monoclonal antibody therapies include Nivolumab and Pembrolizumab (anti-PD-1), Atezolizumab and Durvalumab (anti-PD-L1), Ipilimumab (anti-CTLA-4) and combination Nivolumab and Ipilimumab (anti-PD-1 + anti-CTLA-4). Agonist monoclonal antibody therapies are CP-870,893 (anti-CD40), Urelumab and Utomilumab (anti-4-1BB). Small molecule inhibitors targeting IDO1 include Epacadostat.

Unfortunately, the efficacy of these agents is associated with autoimmune-like inflammatory side effects, termed immune-related adverse events (irAEs) in a large proportion of patients [6,7,8]. These novel drug side effects manifest as tissue destructive immune-mediated toxicity, which can affect any organ [9]. Common irAEs include colitis, dermatitis and hepatitis [7,8,9,10] and are classified according to the Common Terminology Criteria for Adverse Events (CTCAE) grading system ranging from 1–5 in ascending severity, with 5 being fatality. Hepatotoxicity is among the most frequently encountered irAEs (see Figure 1 for main immunotherapy regimens and their targets associated with hepatotoxicity) [7,10,11,12,13]. The development of drug-related hepatoxicity frequently requires the interruption or permanent cessation of immunotherapy. While the immunopathogenesis is not completely understood, there is emerging evidence for the involvement of myeloid cells, in particular monocytes and macrophages [14,15,16,17].

In this review we will summarise our current understanding of the maintenance of immune tolerance in liver homeostasis, recognised pathways to liver inflammation when tolerance is broken, the role of immunotherapy in mediating breakdown of tolerance and the involvement of myeloid cells in related hepatotoxicity.

## 2. Liver Function during Homeostasis

The liver is uniquely perfused with mixed arterio-venous blood. The dual blood supply exposes the liver to high levels of microbial and dietary products coming from the gastrointestinal tract via the portal vein. This exposure, coupled with tissue remodelling and metabolic functions of the liver, necessitates a distinct immune privileged environment. In order to prevent excessive activation of immune cells triggered by this tonic exposure, the liver is biased towards immune unresponsiveness [24,25].

### Mechanisms of Liver Immune Tolerance

During homeostasis, tolerance suppresses the initiation of inflammation against self and non-self antigenic proteins [26]. Hepatic tolerance is mediated by various suppressive mechanisms, including reprogramming of immune cell function and the presence of immunosuppressive cells including regulatory T cells (Tregs), cytokines (e.g., interleukin 10 (IL-10), transforming growth factor β (TGFβ)) and inhibitory receptor/ligand interactions (e.g., PD-1/PD-L1) [27,28,29]. The liver is enriched with a variety of liver-resident and circulating myeloid cells including infiltrating monocytes, monocyte-derived macrophages (MoMF), liver-resident Kupffer cells (KC) and neutrophils [30,31]. These cells, together with lymphocytes, play crucial roles in promoting immune tolerance during homeostasis and liver inflammation following injury or infection (see Table 1 for key features).

As the liver is exposed to a constant presence of low levels of microbial peptides such as lipopolysaccharide (LPS) coming from the gut microbiome, parenchymal and non-parenchymal cells are often refractory to stimulation by toll-like receptor 4 (TLR4) [41,42,43]. This state is termed ‘endotoxin tolerance’. Endotoxin tolerance leads to a fairly weak response of hepatocytes to TLR stimulation [44,45]. Liver-resident Kupffer cells (KCs), which comprise approximately 80% of the body’s tissue-resident macrophages and 35% of non-parenchymal liver cells [46], produce predominantly anti-inflammatory cytokines (e.g., IL-10 and TGFβ) in response to low-level LPS exposure [47,48].

To further promote hepatic tolerance, KCs downregulate co-stimulatory molecules such as CD80/86 and have reduced expression of major histocompatibility complex (MHC) molecules required for the activation of the adaptive immune compartment [41]. Although they still express low levels of MHC molecules for T cell activation, the reduced levels of co-stimulatory molecules lead to an incomplete activation of T cells. This subsequently leads to an initial proliferation of T cells followed by clonal exhaustion and anergy, characterised by the upregulation of negative regulatory immune checkpoints (e.g., T cell immunoglobulin and mucin domain 3 (TIM-3), PD-1 and CTLA-4), and ultimately apoptosis [49,50]. In contrast, Tregs constitutively express immune checkpoint receptors and their interaction with ligands induces Treg activation [51,52]. Tregs are essential for maintenance of peripheral tolerance and they enhance the immunosuppressive milieu of the liver either via cell-to-cell contact (e.g., CTLA-4/CD80 and CD86 interaction) or through the secretion of the suppressive cytokines (e.g., IL-10 and TGFβ) [53,54]. Even though Tregs play a major role in promoting liver tolerance, research by Kido et al. demonstrated the importance of PD-1/PD-L1-mediated immune regulation in the liver in the absence of Tregs [55]. They report that, in experimental autoimmune hepatitis (AIH), liver inflammation could only be induced following neonatal thymectomy for the depletion of Tregs in combination with genetic deletion of PD-1 [55]. This concomitant loss of Tregs and PD-1 regulation was characterised by liver infiltration of autoreactive CD4^+^ and CD8^+^ T cells and severe hepatitis and the progression to fatal AIH [55].

While liver-resident cells such as KC and the hepatic endothelium constitutively express ligands for inhibitory immune receptors (e.g., PD-L1), receptor expression is usually induced in effector cells by the hepatic environment and engagement with their ligands leads to further suppression of immune function [49,56,57]. PD-L1 expression on liver sinusoidal endothelial cells has been shown to be required for the local induction of CD8^+^ T cell tolerance [58]. In 2004, Dong et al. showed that genetic deletion of PD-L1 in mice causes the spontaneous infiltration and accumulation of previously activated CD8^+^ T cells within the liver [59]. Dong et al. further showed a rapid and more severe progression of liver injury during a model of T cell-mediated hepatitis using Concanavalin A in PD-L1 knockout mice compared to wild-type mice [59]. This suggests a potential role of PD-L1 in the deletion of CD8^+^ T cells to protect the liver from activated cytotoxic T cells.

Maintenance of this balance between immune activation and tolerance is essential for a healthy hepatic environment and its dysregulation can cause tissue damaging inflammatory responses.

## 3. Hepatoxicity Related to Cancer Immunotherapies

**Figure 2:**
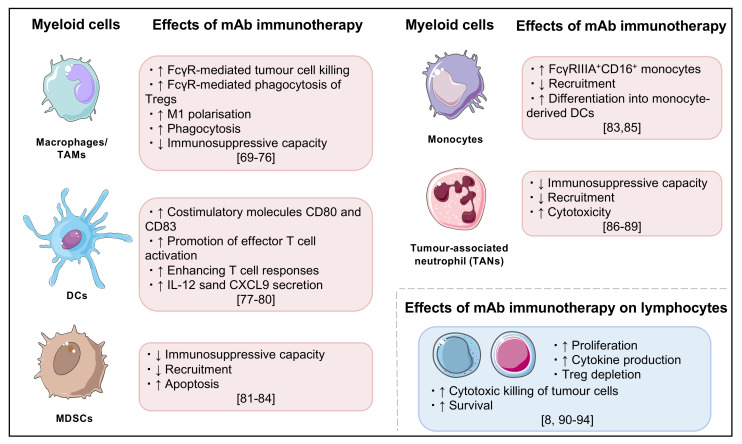
Summary of key effects of monoclonal antibody cancer immunotherapy, particularly on myeloid cells and lymphocytes [69,70,71,72,73,74,75,76,77,78,79,80,81,82,83,84,85,86,87,88,89,90,91,92,93,94].

Improved understanding of regulatory and activating pathways and their role in cancer immunology has led to a therapeutic breakthrough in oncology treatment [60,61]. The use of immunotherapy generally aims to re-programme immune cells to stimulate anti-tumour immune responses (see Figure 2 for key effects of monoclonal antibody cancer immunotherapy on myeloid and lymphoid cells) [1,2,3,62]. However, the manipulation of the balance between immune activation and suppression/tolerance may result in off-target initiation of inflammation in a number of organs, including the liver (Table 2) [1,7,13,22,63]. Liver toxicity during checkpoint inhibitor treatment generally presents as an asymptomatic elevation in serum liver enzymes, typically alanine transaminase (ALT) and aspartate transaminase (AST) levels indicative of hepatocellular damage, though rarer biliary patterns of injury have been described [13,64,65,66]. The clinical course can range from mild, self-limiting inflammation to fulminant hepatic failure and death. The main histological pattern of liver injury is lymphocyte-rich lobular inflammation with spotty or confluent necrosis, hepatocyte apoptosis, ballooning degeneration of hepatocytes and immune aggregates which may form ring granulomas [13,66,67,68].

This form of liver toxicity is mechanistically distinct from other forms of DILI, as it is a result of global immune reprogramming, that in a proportion of individuals leads to hepatocyte-targeted, immune-mediated toxicity [95], as opposed to direct hepatocellular damage by drug or their metabolites. CPI-induced hepatitis is also pathologically distinct from idiopathic AIH. In CPI-induced hepatitis, very few patients test positive for antinuclear antibodies or display hypergammaglobulinemia and discontinuation of cancer therapy and administration of immunosuppressants usually resolves liver inflammation [13]. Histologically CPI-induced hepatitis can be distinguished from both AIH and DILI [67], suggesting that immunotherapy induced hepatoxicity differs pathologically as well as clinically from other recognised forms of liver injury.

### 3.1. Breaking Hepatic Tolerance

As discussed above, due to the liver’s high tonic exposure to dietary and gut microbial antigen, it contains an abundance of immunosuppressive molecules, in particular immune checkpoints, which are constitutively expressed by myeloid cells in the liver [31,47]. These regulatory molecules are important in immune regulation and maintenance of tolerance and protect the liver from spontaneous induction of inflammation by non-pathogenic cues [47,99,100,101,102]. Under inflammatory conditions, this tolerance can be overcome but requires a high threshold of antigenic, TLR and cytokine stimulations to induce robust immune responses [103]. Following an inflammatory response, these immunosuppressive pathways in the liver are crucial for the resolution of inflammation and promotion of tissue repair [104]. Thus, blocking these pivotal regulatory pathways has the potential to lower the high activation threshold of the liver and render it susceptible to an acute inflammatory response. While the exact mechanism of hepatoxicity related to immunotherapy is incompletely understood, increasing evidence describes a role of myeloid cells and in particular monocytes and macrophages in the disease pathogenesis, either by directly causing tissue damage or by the activation of cytotoxic cells (Table 3).

### 3.2. Checkpoint Inhibitors (CPIs)

CPI-induced hepatitis represents one of the most common irAEs associated with CPI therapy. CPIs target immune cell checkpoints such as CTLA-4, PD-1 and PD-L1 that are not only important for T cell regulation, but also control innate inflammatory responses [107,108]. Approximately 5–10% of patients treated with single-agent CPIs and 25–30% on combination CPI therapy [7,11,12,95] experience CPI-induced hepatitis and it usually occurs within one and three months of treatment [109].

Our group recently reported a description of the peripheral and intra-hepatic immune phenotype of monocytes/macrophages and CD8^+^ T cells in patients with CPI-induced hepatitis [14] and provided evidence for the involvement of myeloid cells in the pathogenesis. In CPI-induced hepatitis, circulating classical monocytes were expanded and showed an activated, tissue homing phenotype (CD163^high^CCR2^high^CCR7^low^), in which the proportion of classical monocytes and CD163 expression correlated positively with disease severity. This was accompanied by high levels of soluble CD163 in sera from patients with CPI-induced hepatitis, which has been shown to be a biomarker of monocyte/macrophage activation in other acute liver injury syndromes [110,111,112]. The transcriptional profile of circulating monocytes from patients with CPI-induced hepatitis demonstrated an increased expression of genes associated with activation and survival factors and reduced expression of negative regulators. Monocytes from patients with CPI-induced hepatitis showed further elevation of activation (CD40^high^CD163^high^) and increased secretion of pro-inflammatory cytokines (IL-1β, IL-6, IFNγ, IL-12p70, TNFα), once differentiated into macrophages in vitro.

**Figure 3:**
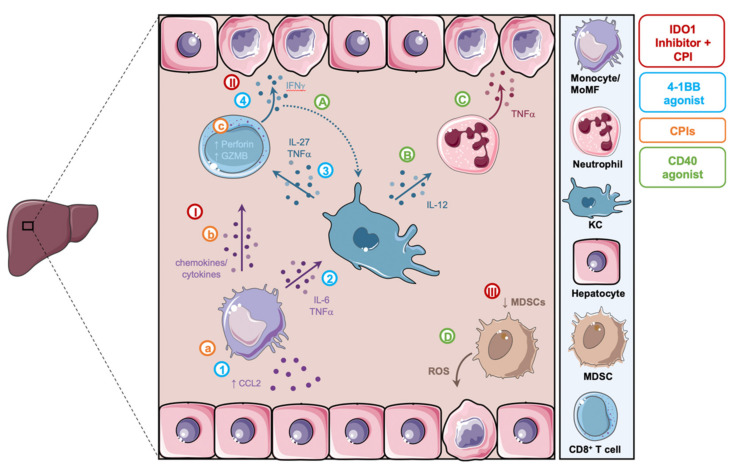
Summary of proposed mechanistic models of hepatoxicity related to cancer immunotherapy. (A,B) Following CD40 agonism, KCs sense IFNγ secreted by CD8^+^ T cells and activate neutrophils in an IL-12 dependent manner. (C) These neutrophils in turn secrete TNFα leading to hepatocyte damage. (D) CD40 agonism reprograms MDSCs and causes the release of ROS and hepatocyte death. (a–c) CPIs induce the activation of monocytes and highly cytotoxic CD8^+^ T cells and lead to the formation of inflammatory aggregates associated with hepatotoxicity. (I–III) IDO1 inhibitors in combination with CPIs lead to the activation of CD8^+^ T cells by MoMFs, their secretion of IFNγ and subsequent hepatocellular injury, as well as the reduction in MDSCs. (1–4) 4-1BB agonism leads to the activation and CCL2/CCR2-dependent liver recruitment of MoMFs. MoMFs promote the activation of KCs, which in turn activate tissue damaging IFNγ secreting CD8^+^ T cells. MDSC, myeloid-derived suppressor cells; ROS, reactive oxygen species; KC, kupffer cells; MoMF, monocyte-derived macrophages; CPIs, checkpoint inhibitors; GZMB, granzyme B.

The CCR2^+^ tissue-homing inflammatory phenotype of circulating monocytes correlated positively with activation markers of cytotoxic CD8^+^ T cells [14]. Parallel to these systemic changes, liver biopsies from CPI-induced hepatitis patients show focal immune aggregates composed of cytotoxic granzyme B^+^CD8^+^ T cells co-localising with CD163^+^ and CCR2^+^ expressing MoMFs. While further investigations are necessary, the presence of CCR2^high^ monocytes in blood and CCR2^+^ MoMF in liver biopsies suggests monocyte recruitment to the liver from the circulating monocyte pool may be, together with their interaction with cytotoxic CD8^+^ T cells, mechanistically important in the pathogenesis of CPI-induced hepatitis (Figure 3a–c).

### 3.3. Agonistic Anti-CD40

Another immunotherapy that has received recent attention is agonistic anti-CD40 treatment. CD40 is expressed on antigen presenting cells (APCs) and stimulates CD8^+^ T cell activation and pro-inflammatory Th1-polarisation [113,114]. In response, T cells are able to overcome tumour-induced tolerance and produce IFNγ and IL-12 for the initiation of anti-tumour immunity [15,115,116]. However, several clinical trials reported the development of severe adverse events, most commonly cytokine release syndrome and hepatotoxicity, in response to agonistic anti-CD40 therapy [3,10,23,117]. Here, hepatoxicity, similarly to other forms of immunotherapy-induced liver injury, is associated with increased liver enzymes such as ALT and AST [10].

Early research reported a myeloid-derived suppressor cell (MDSC) associated phenotype in CD40 agonist-induced hepatoxicity [105]. CD40 plays an important role in the maturation of immunosuppressive MDSCs. In murine livers and in patients with HCC, accumulations of tumour-induced MDSCs have been observed [118,119]. Moreover, hepatic MDSCs have also been associated with the generation of hepatic metastases [120]. The treatment with CD40 agonist monoclonal antibodies activates tumour-induced myeloid cells and reduces the suppressive function of murine and human MDSCs [105]. Medina-Echeverz et al. proposed that anti-CD40 treatment caused reprogramming of CD11b^+^Gr-1^+^ MDSCs to a proinflammatory phenotype, lacking a suppressor function which ultimately leads to the release of reactive oxygen species (ROS) and hepatocyte death (Figure 3D) [105].

In contrast, recent animal studies investigating the underlying mechanism of anti-CD40 induced hepatoxicity reported a complex interplay between Th1 and various subsets of myeloid cells that dictate hepatotoxicity [15,64]. For example, in 2020 Bonnans et al. showed that CD40 agonist induced an inflammatory network of TNFα, IFNγ and IL-12, in which only deficiency in IL-12 was protective of liver injury and led to the decreased activation and frequency of CD11b^+^CD14^+^F4/80^+^MHCII^+^ hepatic macrophages [64]. Similarly, in 2021 Siwicki et al. show the induction of pathology by IFNγ and IL-12 in tumour free tissues, which was dependent on macrophages and neutrophils [15]. In contrast, IL-12 and IFNγ producing DC and cytotoxic CD8^+^ T cells mediated anti-tumour immunity but were not necessary for tissue pathology. In the liver, KCs were able to sense IFNγ secreted by T cells and in turn produced IL-12. Using transgenic mice treated with anti-CD40 in which KCs lack the receptor for IFNγ (*Clec4f-cr^+/0^ Ifngr1^fl/fl^*), the authors showed that IL-12 production and liver necrosis were nearly diminished. They further showed that the IL-12 response of KC mediated by IFNγ acted as a positive feedback loop promoting local IFNγ production. IFNγ sensing subsequently induced the increased presence and *Tnf* expression of tissue damaging neutrophils in the liver (Figure 3A–C). Neutrophils were the main source of *Tnf* as they contributed approximately 92% of all *Tnf* expression in the inflamed liver. Neutrophil and TNFɑ neutralisation in mice treated with CD40 agonist led to the protection from liver necrosis and inflammation. Siwicki et al. further demonstrated similarities between these findings to the pathology of CPI-induced hepatitis. They describe neutrophil liver infiltrates to be associated with severity of inflammation in human CPI-induced hepatitis. Mice treated with combination anti-CTLA-4 and anti-PD-1 present with elevated levels of IL-12 in tumour free tissues, an activated MHCII^high^ phenotype of KCs and an increase in liver neutrophils.

In this study, neutrophil based interventions were able to suppress CD40 agonist associated liver damage without negatively impacting on the tumour response [15]. Not only may targeting neutrophils have the potential to treat hepatoxicity in this context and potentially other immunotherapy related hepatotoxicities, but neutrophil based therapies are currently being trialled as cancer therapy, making this an attractive adjunct to CD40 agonism [121,122].

### 3.4. 4-1BB Activation

4-1BB (CD137) in an activation-induced costimulatory receptor and is expressed by a wide range of activated lymphocyte and myeloid subsets [62]. The interaction with its ligand stimulates activation of these cells and promotes CD8 driven anti-tumour responses [123]. However, despite the effectiveness of this treatment approach, the activation of the 4-1BB pathway was associated with dose-limiting severe hepatocellular injury in pre-clinical trials and no trial has advanced beyond early phase II [62].

Research by Bartkowiak et al. showed that 4-1BB agonist-associated hepatotoxicity is not triggered by activated CD8^+^ T cell responses but is initiated through the activation of KCs and their secretion of TNFα and IL-27, which in turn promoted the cytotoxic function of CD8^+^ T cells and hepatocyte damage [17]. They show that 4-1BB activated bone marrow-derived monocytes home to the liver and cause an inflammatory environment which stimulated the upregulation of 4-1BB in KCs. KCs then respond to 4-1BB agonistic therapy by increasing their antigen presentation capacity (MHC II upregulation) and producing IL-27 for the attraction of CD8^+^ T cells. This leads to the activation of CD8^+^ T cells with elevated IFNγ secretion and ultimately local hepatocyte damage (Figure 3 1–4). In the pathogenesis, CD8^+^ T cells directly mediate tissue injury, as mice deficient in CD8^+^ T cells are protected from hepatotoxicity. However, the activation of CD8^+^ T cells is highly dependent on the presence of KC-derived IL-27. They further found that CCR2^−/−^ mice, which are deficient in bone marrow-derived monocytes, were also protected from hepatotoxicity but showed an intact CD8 anti-tumour response. The authors speculate that 4-1BB activated monocytes initiate an immune cascade to trigger off target liver injury and that CCR2 inhibitors are a potential target to treat liver inflammation in this condition.

### 3.5. Combined Agents

The combination of immunotherapeutic agents has shown to be more effective than single agent therapy and to improve overall survival [2,124,125]. For example, in metastatic melanoma, five-year overall survival rates were increased to 52% in Ipilimumab and Nivolumab combination therapy [1]. In contrast, single agent CPI treated patients showed an overall survival of 44%. Moreover, studies showed that combining CPIs with agonists of immune stimulatory molecules as well as various other inhibitors (e.g., small molecules) has beneficial effects in boosting the anti-tumour response [16,17,106].

#### 3.5.1. Checkpoint Inhibitor Combination Therapy

While the combination of CPIs (e.g., Ipilimumab and Nivolumab) was associated with increased survival, the frequency and severity of CPI-induced hepatitis was also higher in patients on dual therapy [1]. In fact, up to 15% of those patients develop high grade hepatitis (grade 3–4, defined by an ALT level of >5 or >20 times the upper limit of normal (ULN)) [11,12]. In contrast, the development of severe, grade 3–4 hepatitis in patients on single therapy is less frequent, with an incidence rate of 1–2%.

The histological features and peripheral immune phenotype associated with anti-CTLA-4 and/or anti-PD-1 mediated liver injury in CPI-induced hepatitis are also distinct [13,126]. For example, liver biopsies showed a more equal CD4:CD8 ratio following single agent anti-PD-1 therapy compared with combination CPI [13]. This could also be observed in the circulation of patients with CPI-induced hepatitis [14]. All treatment regimens were described to have inflammatory liver infiltrates that consisted predominantly of lymphocytes and macrophages; however, the occurrence of those immune aggregates is particularly pronounced in dual agent therapy containing anti-CTLA-4 and anti-PD-1 CPI [13,127]. In contrast, the phenotype of monocytes/macrophages in CPI-induced hepatitis were similar between single and dual agent CPI [14].

#### 3.5.2. Small Molecule Indoleamine 2,3-Dioxygenase 1 (IDO1) Inhibitors and CPIs

IDO1 is a cytosolic enzyme involved in the suppression of cytotoxic cells such as CD8 T cells and natural killer (NK) cells by depletion of tryptophan and the promotion of regulatory T cells, MDSCs and tumour-associated macrophages (TAMs) [128]. In preclinical studies, IDO1 was shown to be involved in the tumour escape from immune surveillance and its activation in human cancers was associated with poor prognosis [129]. Activation of IDO1 can be observed in tumour cells themselves as well as stromal and vascular cells and innate immune cells [130]. The suppressive tumour microenvironment recruits cells expressing IDO1, which in turn recruit and tolerise further immune cells [130]. This created a positive feedback loop for the reprogramming of cells and exacerbating a suppressive milieu. Due to this role in immune-oncology, small molecule inhibitors of IDO1 present a promising concept in cancer therapies [4].

In the liver, hepatic stellate cells were shown to promote tolerogenic DCs by inducing IDO1 expression, enhancing hepatic tolerance [131]. In contrast, in *Ido1* deficient mice (*Ido1^−/−^*), inflammatory stimuli within the liver can overcome the suppressive environment and induce liver inflammation, suggesting a lowered threshold for activation [132,133]. IDO inhibitors such as Epacadostat are currently being trialled as cancer treatment, in particular in combination with CPIs [4]. Murine studies on the use of an IDO1 inhibitor with CPIs demonstrated the development of hepatocyte injury and liver infiltration of primarily CD8^+^ T cells with a reduction in MDSC frequencies (Figure 3 III) [106]. In turn, the number of liver infiltrating MoMF was increased [16]. Similarly to other drug related hepatoxicities, CPI+IDO1 inhibitor induced liver injury was abolished when CD8^+^ T cells were depleted [16]. Single cell and bulk RNA sequencing of these mice revealed that a central role of IFNγ in the disease pathogenesis and MoMF promoting this pro-inflammatory T cell response (Figure 3 I,II). Interestingly, this study also reported on CPI combination therapy together with 4-1BB agonism, which has a similar expression profile [16].

#### 3.5.3. Immunotherapy in Combination with Conventional Chemotherapeutics

To further enhance the effectiveness of immunotherapy and to promote anti-tumour responses in immunotherapy refractory patients, numerous clinical trials are currently exploring immunotherapy combined with chemotherapy [134,135,136,137,138,139,140,141]. Certain chemotherapy agents can be immunostimulatory and increase antigenicity of cancer cells leading to the activation of effector cells such as DCs, M1-like TAMs and cytotoxic cells, and depletion of immunosuppressive cells including MDSCs and Tregs [142,143,144,145,146,147]. This is particularly mediated by agents inducing immunogenic cell death of malignant cells, during which dying cells accumulate nucleic acids in their cytosol and release cytoplasmic and nuclear proteins in their extracellular space to activate the innate and subsequently adaptive immune compartment [148,149]. These agents include, for example, cyclophosphamide, dactinomycin, gemcitabine and cisplatin [149]. Preclinical and clinical studies investigating chemotherapy in combination with anti-PD-1 and anti-CTLA-4 checkpoint inhibitors show promising results with higher response rates and overall survival [142]. In addition, chemotherapies were also demonstrated to promote the upregulation of PD-L1 on various cells such as cancer cells and TAMs [150,151]. Combination of those therapies with anti-PD-(L)1 treatment could further increase anti-tumour responses in patients [152]. While the majority of trials are still ongoing, the increased risk of hepatotoxicity has been associated with the use of combination therapy in a few studies (Table 4) [134,135,136,137,138,139,140,141]. A recent meta-analysis by Guo et al., assessing the risk of hepatoxicity following different treatment regimens involving anti-PD-(L)1 and chemotherapy, showed an elevated risk of all-grade and high-grade hepatitis with use of anti-PD-(L)1 with and without chemotherapy, compared to chemotherapy alone [153]. Thus far, the role of myeloid cell involvement in this is unclear and warrants further investigation.

### 3.6. In Vivo Experimental Models of Immune-Related Adverse Events

Various studies are aiming to establish clinically relevant murine models for the investigation of the underlying immunological mechanisms of liver irAEs and to lay the foundation for in vivo testing of therapeutic agents. One of the major challenges to date is that mice do not spontaneously develop hepatic inflammation in the absence of checkpoint signalling, either through blockade (e.g., anti-CTLA-4, anti-PD-1) or genetic deletion (e.g., PD-1^−/−^) [17,104,106].

In an effort to develop a murine model of drug-induced liver injury (DILI), Metushi et al. reported the development of more severe and persistent DILI when hepatic tolerance is broken by interruption of CPI signalling [104,106,154]. In PD-1^−/−^ mice, the co-treatment of the hepatotoxin Amodiaquine with anti-CTLA-4 resulted in liver infiltration of macrophages and cytotoxic CD8^+^ T cells and the formation of immune cell aggregates in areas of necrosis [104]. This evidence suggested that breakdown of immune checkpoints, normally mediating hepatic immune tolerance mechanisms, promotes liver damage caused by other triggers. This need for a priming event for the initiation of hepatic inflammation in the context of immunotherapy seems to be a common feature in murine models [15,16,17,106] and raises questions about the induction of human pathology. 

In 2021, Adam et al. showed that treatment with anti-CTLA-4 + anti-PD-1 in tumour bearing mice from a genetic background (*B6/lpr* mice) prone to autoimmune responses, induced multiple organ irAEs [155]. Using this model, they demonstrated that most effective anti-tumour responses in mice were associated with the development of irAEs. They postulate that the reduction in tumour size could be used as a predictive marker for irAEs. In fact, early data from patients with melanoma and lung cancer shows correlations of anti-tumour responses following CPI therapy with irAE severity [156,157,158]. Adam et al. further showed that prednisolone treatment in this model prevented the development of irAEs in mice treated with combination CPI [155]. However, it also demolished anti-tumour immunity. While limited due to the mice’ genetic susceptibility to autoimmunity, this informative study shows the importance of establishing effective murine models to inform early detection and treatment regimens of human irAEs.

## 4. Future Perspective

The management of immune-mediated hepatitis in the context of cancer immunotherapies, in a way that does not compromise the anti-tumour response, presents a clinical challenge. New technologies such as single cell RNA sequencing (scRNAseq) make it possible to study highly heterogeneous tumour cells, the immune landscape of the tumour microenvironment and classify new immune subpopulations [159,160,161,162,163]. Such techniques are proving crucial to identify effective diagnostic and prognostic biomarkers, develop new tumour immunotherapy and unravel the complexity of immune interactions during drug toxicity [160,161,162,164,165,166,167]. Novel prediction platforms such as Beyondcell using scRNAseq datasets and drug response profiles have the potential to indicate targetable pathways with very high response rates [168]. Moreover, machine learning algorithms utilising irAE datasets collected from patient symptom questionnaires and Common Terminology Criteria for Adverse Events (CTCAE) have been shown to predict the presence and onset of irAEs with high accuracy and have the potential to aid early detection of irAEs [169]. While early detection systems and biomarkers are important factors for the safety of immunotherapy in patients, the development of alternative treatment strategies that do not require cessation of cancer treatment nor negatively impact the anti-tumour response are paramount. Understanding the underlying mechanisms of these hepatoxicities will inform as to the type and timing of immune-based interventions to resolve liver toxicity. Research to date has demonstrated that targeting myeloid cells such as monocytes and neutrophils presents a promising approach. Although evidence across a number of agents and models suggests that lymphocytes contribute to hepatoxicity, their inhibition would significantly compromise anti-tumour immunity [170,171,172,173]. In contrast, targeted inhibition or depletion of involved myeloid subsets may not directly affect T cell licensing for anti-tumour responses but has the potential to improve hepatotoxicity significantly.

## Figures and Tables

**Table 1:** Key features of myeloid subsets within the liver.

Hepatic Myeloid Subset	Key Features	References
Monocytes/ Monocyte-derived macrophages (MoMF)	- Inflammatory: liver infiltration of monocytes following injury, differentiation into inflammatory macrophages, mediate tissue damage - Restorative: resolution of inflammation, tissue repair	[32,33]
Kupffer cells (KC)	- Maintain a tolerogenic environment - Microbial clearance - Uptake of debris during tissue damage - Facilitators of inflammatory response (cytokine/chemokine secretion)	[34,35]
Myeloid-derived suppressor cells (MDSC)	- Suppress immune responses	[36]
Neutrophils	- Tissue healing - Host defense	[37,38]
Dendritic cells (DC)	- Tolerogenic at steady-state - Pro-inflammatory during injury - Promotion of adaptive immune responses	[39,40]

**Table 2:** Prevalence of hepatic irAEs in major immunotherapy clinical trials.

Immunotherapy Regimen	Any Grade, % (Number of Patients)	Grade 3–5, % (Number of Patients)	Reference
Anti-PD-1/ Anti-PD-L1	1.8 (5/277) 1.8 (9/509) 8 (25/313) 16.1 (46/286)	1.8 (5/277) 1.4 (7/509) 3 (9/313) 4.2 (12/286)	Robert et al., 2015 [18] Eggermont et al., 2018 [19] Larkin et al., 2019 [1] Herbst et al., 2020 [96]
Anti-CTLA-4	1.2 (3/256) 26.4 (19/72) 3.8 (5/131) 15.5 (9/58) 7 (23/311)	0.4 (1/256) 0 (0/72) 0 (0/131) 10.3 (6/58) 2 (5/311)	Robert et al., 2015 [18] Wolchok et al., 2010 [20] Hodi et al., 2010 [21] Weber et al., 2009 [97] Larkin et al., 2019 [1]
Combination anti-CTLA-4 and anti-PD-1	23 (12/53) 17.6 (55/313) 22.3 (21/94) 33 (103/313)	15 (8/53) 8.3 (26/313) 10.6 (10/94) 20 (62/313)	Wolchok et al., 2013 [63] Larkin et al., 2015 [22] Postow et al., 2015 [98] Larkin et al., 2019 [1]

**Table 3:** Summary of the effect of different classes of immunotherapy on hepatic myeloid cells and their crosstalk with other subsets.

Immunotherapy Mechanism	Direct Effect on Hepatic Myeloid Cells and Their Crosstalk with Other Subsets	Reference
Checkpoint inhibitors (e.g., anti-PD-(L)1, anti-CTLA-4, anti-PD-1 + anti-CTLA-4)	Activation and liver homing of monocytes/MoMF, monocyte interaction with CD8^+^ T cells	Gudd et al., 2021 [14]
Agonistic anti-CD40	Activation of KCs, recruitment and activation of neutrophils, reduced suppressive capacity of MDSCs	Medina-Echeverz et al., 2015 [105] Siwicki et al., 2021 [15] Bonnans et al., 2020 [64]
4-1BB activation	Liver homing of monocytes, activation of KCs, activation of CD8^+^ T cells by KCs	Bartkowiak et al., 2018 [17]
IDO1 inhibitors in combination with CPIs (e.g., anti-CTLA-4, anti-PD-(L)1, anti-CTLA-4 + anti-PD-1)	Activation of MoMF, promotion of CD8^+^ T cell activation by MoMF, reduction of MDSCs	Affolter et al., 2019 [106] Llewellyn et al., 2021 [16]

**Table 4:** Reported hepatoxicity related to cancer immunotherapy combined with chemotherapy.

Immunotherapy	Chemotherapy	Reference
Ipilimumab (anti-CTLA-4)	Dacarbazine	Robert et al., 2011 [134]
Pembrolizumab (anti-PD-1)	Carboplatin, pemetrexed, nab-paclitaxel	Gandhi et al., 2018 [141] Langer et al., 2016 [135] Paz-Ares et al., 2018 [136]
Atezolizumab (anti-PD-L1)	Carboplatin, nab-paclitaxel, etoposide	Horn et al., 2018 [137] Schmid et al., 2018 [138] Socinski et al., 2018 [139] West et al., 2019 [140]
